# Variable Expressivity in Type 2 Familial Partial Lipodystrophy Related to R482 and N466 Variants in the LMNA Gene

**DOI:** 10.3390/jcm10061259

**Published:** 2021-03-18

**Authors:** David Araújo-Vilar, Sofía Sánchez-Iglesias, Ana I. Castro, Silvia Cobelo-Gómez, Álvaro Hermida-Ameijeiras, Gemma Rodríguez-Carnero, Felipe F. Casanueva, Antía Fernández-Pombo

**Affiliations:** 1 UETeM-Molecular Pathology Group, Department of Psychiatry, Radiology, Public Health, Nursing and Medicine, IDIS-CIMUS, University of Santiago de Compostela, 15706 Santiago de Compostela, Spain; david.araujo@usc.es (D.A.-V.); sofia.sanchez@usc.es (S.S.-I.); silviacobelog@gmail.com (S.C.-G.); alvaro.hermida@usc.es (Á.H.-A.); 2 Division of Endocrinology and Nutrition, University Clinical Hospital of Santiago de Compostela, 15706 Santiago de Compostela, Spain; anaisabel0121@gmail.com (A.I.C.); mrcarnero@alumni.unav.es (G.R.-C.); felipe.casanueva@usc.es (F.F.C.); 3 CIBER Fisiopatología de la Obesidad y la Nutrición (CIBERobn), 28029 Madrid, Spain; 4 Division of Internal Medicine, University Clinical Hospital of Santiago de Compostela, 15706 Santiago de Compostela, Spain; 5 Epigenomics in Endocrinology and Nutrition Group, Epigenomics Unit, Instituto de Investigación Sanitaria de Santiago de Compostela (IDIS), University Clinical Hospital of Santiago de Compostela, 15706 Santiago de Compostela, Spain

**Keywords:** lipodystrophy, Dunnigan disease, *LMNA*, laminopathies, body composition, DXA, variable expressivity

## Abstract

Patients with Dunnigan disease (FPLD2) with a pathogenic variant affecting exon 8 of the *LMNA* gene are considered to have the classic disease, whereas those with variants in other exons manifest the “atypical” disease. The aim of this study was to investigate the degree of variable expressivity when comparing patients carrying the R482 and N466 variants in exon 8. Thus, 47 subjects with FPLD2 were studied: one group of 15 patients carrying the N466 variant and the other group of 32 patients with the R482 variant. Clinical, metabolic, and body composition data were compared between both groups. The thigh skinfold thickness was significantly decreased in the R482 group in comparison with the N466 group (4.2 ± 1.8 and 5.6 ± 2.0 mm, respectively, *p* = 0.002), with no other differences in body composition. Patients with the N466 variant showed higher triglyceride levels (177.5 [56–1937] vs. 130.0 [55–505] mg/dL, *p* = 0.029) and acute pancreatitis was only present in these subjects (20%). Other classic metabolic abnormalities related with the disease were present regardless of the pathogenic variant. Thus, although FPLD2 patients with the R482 and N466 variants share most of the classic characteristics, some phenotypic and metabolic differences suggest possible heterogeneity even within exon 8 of the *LMNA* gene.

## 1. Introduction

Type 2 familial partial lipodystrophy (FPLD2) or Dunnigan disease is a rare autosomal dominant disorder (OMIM #151660) characterized by the loss of adipose tissue in the limbs, trunk, and gluteal region and its accumulation in the face, neck, axillae, interscapular area, labia majora, and abdominal viscera. It occurs after puberty in the case of women and later in men [1,2,3]. In addition, the classic phenotype is often associated with phlebomegaly, muscular hypertrophy, and acanthosis nigricans. Patients with FPLD2 are predisposed to metabolic complications related to insulin resistance [4] and an inability to properly store lipids such as diabetes mellitus (DM), hypertriglyceridemia with resultant pancreatitis, hepatic steatosis, and cardiovascular complications such as premature atherosclerosis or rhythm disturbances [5,6]. Women can also have polycystic ovary syndrome (PCOS) and other fertility problems [7,8,9,10,11].

The pathogenic variants responsible for this syndrome are located in the *LMNA* gene that encodes nuclear lamin A/C on chromosome 1q21-22, and more than 80% of FPLD2 cases are associated to a missense variant affecting the arginine residue at 482 position (p.R482Q, p.R482W, p.R482L) in exon 8 [12,13,14]. However, other *LMNA* pathogenic variants in exon 8 and other exons have also been described [15,16,17,18,19]. The ubiquitous expression of this gene also underlies the broad spectrum of other lamin-related diseases, which share overlapping pathologies and heterogeneous phenotypes, such as Hutchinson–Gilford progeria syndrome, mandibuloacral dysplasia type A, restrictive dermopathy, Emery–Dreifuss muscular dystrophy, limb–girdle muscular dystrophy and atypical Werner syndrome [20]. These laminopathies are of particular interest due to the relatively poor genotype–phenotype correlation. Therefore, different missense variants of the same amino acid residue, or even the same variant, can result in different clinical syndromes [21]. In the case of FPLD2, most patients with the classic phenotype are those who harbor heterozygous missense variants affecting arginine at codon 482 in exon 8 of the *LMNA* gene, while those presenting with other *LMNA* variants in other exons have variable loss of adipose tissue and are considered to have “atypical” FPLD [11,22]. The pathogenic variant N466 is also responsible for FPLD2 and also lies within exon 8 of the *LMNA* gene [17]. However, it is apparently less frequent and, to the best of our knowledge, there are no studies that determine the possible differences in the adipose phenotype and comorbidities of the subjects carrying this variant in comparison with the considered classic variant in the same exon of the gene.

The aim of this study was to compare anthropometric and body composition data, clinical characteristics, comorbidities, and metabolic parameters in FPLD2 patients with two different variants of the *LMNA* gene in exon 8, R482 and N466, in order to investigate the degree of heterogeneity of the disease.

## 2. Materials and Methods

This study was approved by the ethics review panel of the Red Gallega de Comités de Ética de la Investigación (approval code 2017/477) and carried out according to the ethical guidelines of the Helsinki Declaration.

### 2.1. Study Design and Population

This is a retrospective, observational, longitudinal study, carried out in a single center, in which two groups of 47 subjects with molecular diagnosis of FPLD2 from different regions of Spain were studied: one group of 15 patients carrying the p.(Asn466Asp) variant and another group of 32 patients with the R482 variant (30 carrying the p.(R482W) variant and 2 carrying the p.(R482Q) variant) in exon 8 of the *LMNA* gene (see Table 1 for detailed information). Each patient only presented one single pathogenic variant in heterozygosity within the *LMNA* gene. Prepuberal subjects were excluded taking into account that the expected phenotype is not yet fully expressed in these patients. Demographic, anthropometric, body composition, and clinical data, as well as metabolic parameters were compared between both groups.

### 2.2. Anthropometry and Body Composition

Physical examination was performed on all participants in the study. Their height and weight were verified with digital scales and a stadiometer. The skinfolds of the patients with FPLD2 were measured using a Lange skinfold caliper (Cambridge Scientific Industries, Cambridge, MD, USA) by a single examiner. Determination of bone mineral density (BMD), both total and segmental, as well as fat mass (FM) and fat-free mass determination (FFM) was performed using whole-body dual-energy X-ray absorptiometry (DXA) with a Lunar DPX model (GE Healthcare Lunar, Madison, WI, USA). Visceral fat was calculated using a developed software (Core Scan^®^ GE Healthcare, Madison, WI, USA).

### 2.3. Analytical and Clinical Data

Blood samples were taken between 8:00–9:00 a.m. after 12 h overnight fasting. Glucose, creatinine, triglycerides, and total and fractionated cholesterol levels were measured by standardized methods with appropriate quality control and quality assurance procedures. Aspartate aminotransferase (AST), alanine aminotransferase (ALT), and γ-glutamyl transpeptidase (GGT) were determined with enzymatic methods on an ADVIA analyzer (Siemens, Bayer Diagnostics, Tarrytown, NY, USA). Glycated hemoglobin (HbA1c) was measured with ion-exchange high-performance liquid chromatography (Bio-Rad Laboratories Inc., Hercules, CA, USA). Plasma insulin concentrations were determined in duplicate by chemiluminescence using a commercial kit (Nichols Institute, San Juan Capistrano, CA, USA). Plasma leptin levels and C-peptide were determined by enzyme-linked immunosorbent assay (ELISA) (DRG International Inc., Springfield, NJ, USA).

Clinical data were obtained from the electronic medical records of the patients. Diabetes was defined according to the 2020 American Diabetes Association criteria [23]. Polycystic ovary syndrome (PCOS) was diagnosed based on the presence of at least two of the following three criteria: chronic anovulation, clinical and/or biochemical hyperandrogenism, and polycystic ovaries on ultrasound, after excluding other androgen excess or related disorders. Hepatic steatosis was measured using high resolution ultrasound B-mode imaging with a convex transducer (frequency of 3.5–5 MHz).

### 2.4. LMNA Variant Analysis

DNA was prepared from peripheral white blood cells with standard procedures [24]. *LMNA* exons 1–12 and the surrounding intronic sequences were amplified by PCR using primers and conditions available upon request. After purification with ExoSAP-IT^TM^ (Applied Biosystems, Waltham, MA, USA), the PCR products were directly sequenced with the same primers used for amplification and the BigDye Terminator v3.1 Cycle Sequencing Kit (Applied Biosystems). Electropherograms were produced with an ABI 3100 automated sequencing analyzer (Applied Biosystems), and analyzed using DNA Baser (2020, Heracle BioSoft v.5.8.0, www.dnabaser.com, accessed on 17 March 2021).

### 2.5. Statistical Analysis

Data are expressed as the mean ± standard deviation (SD), median, and interquartile range (IQR) or as percentages. Fisher’s exact test was used to compare qualitative variables. The Mann–Whitney test was used to compare a quantitative variable in two groups. The level of significance was set at *p* < 0.05. All statistical analyses were performed with the SPSS 22.0 program (Chicago, IL, USA).

## 3. Results

In this study, 32 patients with the R482 variant (75.0% females, 51.8 ± 18.1 years of age) and 15 patients with the N466 variant (73.3% females, 44.1 ± 19.9 years of age) in exon 8 of the *LMNA* gene, belonging to 15 different families, were compared. Demographic and anthropometric data, as well as body composition determined by DXA, are shown in Table 2. In both groups, about half of the patients presented with the characteristic phenotype of abnormal fat distribution around puberty. All the patients who debuted with lipodystrophy in adulthood, with the exception of two with the R482 variant, were men. The skinfold in the thigh was significantly lower in the R482 group compared to the N466 group (4.2 ± 1.8 mm vs. 5.6 ± 2.0 mm, *p* = 0.002), without differences in the skinfolds of the biceps, triceps, the subscapular and suprailiac regions, and calf, or in the waist and hip perimeters. However, regarding the DXA measurement of fat content, there were no differences in the adipose tissue distribution between both groups in the lower limbs or in other areas, or in terms of FFM. As expected, as far as total and segmental BMD are concerned, there were also no significant differences.

Table 3 summarizes the clinical data of the FPLD2 patients. Metabolic abnormalities associated with insulin resistance were present in the subjects with FPLD2 regardless of the pathogenic variant. Muscle hypertrophy and phlebomegaly were also present in 68.8/73.3% and 81.2/80.0% of the patients with R482/N466 variants, respectively. Pancreatitis was only present in subjects with the N466 variant (20.0% of the cases), which was in accordance with the highest triglyceride levels in this group. DM was present in 31.2% of the R482 cases and in 40.0% of the N466 cases, and hypertension in 28.1% and 30.0% of the R482 and N466 cases, respectively. Two patients with the N466 variant and one patient with the R482 variant manifested nephropathy (the latter requiring dialysis). Retinopathy was present in only one patient with diabetes in each group. Regarding cardiovascular disease, there were no differences in the frequency of ischemic cardiopathy or left ventricular hypertrophy, and no cases of cardiac arrhythmia were observed in either group. FPLD2 patients also did not develop peripheral vascular disease or stroke. PCOS was present in women in both groups without significant differences, and 3 women of the R482 group experienced pregnancy loss.

As for analytical measurements (Table 4), triglyceride levels were significantly higher in subjects with the N466 variant compared to subjects with the R482 variant (177.5 [56–1937] vs. 130.0 [55–505] mg/dL, respectively; *p* = 0.029). Although significant differences were also observed in terms of low-density lipoprotein cholesterol (LDL-C) levels (apparently lower in the N466 group), no differences were observed regarding non-high-density lipoprotein cholesterol (non-HDL-C). Neither were differences found in serum leptin levels nor in the rest of the parameters shown in Table 4.

## 4. Discussion

The present study suggests variable expressivity of the disorder to a mild degree when comparing variants within the same exon of the *LMNA* gene (exon 8, considered to be affected in the “typical” disease) in patients with FPLD2. Thus, although both the R482 and the N466 variants share most of the same classic characteristics, a few differences were observed regarding anthropometry (less severe fat loss in the thighs in patients with the N466 variant according to the skinfold thickness, without other differences in body composition) and metabolic abnormalities (higher triglyceride levels and episodes of pancreatitis in patients with the N466 variant).

Dunnigan disease, or FPLD2, is considered to be one of a group of rare disorders known as laminopathies, which result from pathogenic variants in the *LMNA* gene (primary laminopathies), or other genes that influence lamin processing or the proper functioning of lamin A on chromatin, such as *ZMPSTE24* gene variants in type B mandibuloacral dysplasia [25], or *BANF1* gene variants in Nestor–Guillermo progeria syndrome [26], respectively (secondary laminopathies). The most fascinating peculiarities of laminopathies are their complex genotype–phenotype correlation and clinical heterogeneity [20,27], which is also expressed in overlapping syndromes, characterized by the coexistence of lipodystrophy with skeletal myopathy, cardiomyopathy, neuropathy, and/or premature ageing stigmata, giving rise to the concept of monogenic multi-system diseases [20,28].

Two main mechanisms have been suggested to explain how these *LMNA* variants mediate the observed diseases: the structural model, which proposes that lamin variants impair the ability of the nuclear lamina to maintain nuclear integrity, and the gene expression model, which suggests that lamin variants impair its capacity to interact with chromatin and/or transcriptional regulators central to cellular fate [29,30,31]. In this sense, FPLD2-causing variants probably lead to pathogenicity by disrupting gene regulation, although it has also recently been proposed that abnormal fat distribution could be related to impaired white adipocyte turnover and the failure of adipose tissue browning, both linked to impaired autophagy [32]. It has already been described in previous studies that FPLD2 patients with a missense variant affecting exon 8 are considered to have the classic disease, whereas FPLD2 patients with variants in other positions are considered to have atypical disease, with a unique adipose tissue distribution, which often constitutes a real diagnostic challenge [11,33]. However, there are no data comparing different variants within exon 8, or specific studies that determine if there are still significant clinical or metabolic differences between the classic variant (R482) and other variants in the same exon of the *LMNA* gene, such as N466.

Regarding detailed evaluation of body composition, in this study, adipose tissue distribution according to anthropometry revealed less fat loss in the thighs of patients with the N466 variant in comparison with the classic R482 variant. However, FM measured by DXA showed no differences in the lower limbs, comprising not only the proximal extremities but also their distal part. This reduced loss of adipose tissue in the thighs has also been previously described in patients with atypical FPLD2 due to the R582H variant in exon 11 [11]. In contrast, no differences in fat distribution in the biceps, triceps, subscapular and suprailiac regions, and calf, or in the areas evaluated by DXA, including visceral fat, were found in our study. As far as BMD is concerned, we have already described that, unlike in other types of laminopathies in which the bone is affected, in the case of FPLD there are no differences in BMD [34].

As expected, both pathogenic variants were associated with insulin resistance and various metabolic abnormalities. Patients with the N466 variant showed higher serum triglyceride levels and, accordingly, acute pancreatitis was only present in these subjects. On the contrary, patients with the R482 variant showed higher LDL-C concentrations. However, no differences were observed regarding non-HDL-C between both groups. This suggests that other lipoproteins that are part of non-HDL-C, unlike LDL-C, are likely to be elevated in the case of patients with the N466 variant (lipoproteins containing apolipoprotein B such as very low-density lipoprotein [VLDL] and chylomicron remnants). Thus, it is known that, in subjects with elevated plasma triglyceride levels, the prolonged residence time of VLDL particles allows them to accumulate cholesteryl ester by transfer from other lipoproteins, and remnant lipoprotein species are formed [35]. In addition, both the plasma concentrations of non-HDL-C and LDL-C are strongly associated with the long-term risk of cardiovascular disease [36]. In fact, the presence of ischemic cardiopathy was not a differential finding in either of the groups of subjects studied.

The main limitation of this study is its sample size, which is in accordance with the fact that this disease is considered to be ultra-rare given the estimates of the prevalence of FPLD [37] and the fact that our research has focused on just two specific variants on the *LMNA* gene. However, we believe that this can also be considered to be a strength, taking into account that, to the best of our knowledge, there is no previous research in this specific field with a sufficient number of FPLD2 patients to carry out studies which are more than descriptive.

In conclusion, although patients with FPLD2 due to the *LMNA* pathogenic variants R482 and N466 share most of the classic characteristics of the disease, there are certain phenotypic and metabolic differences, which suggest the possible heterogeneity of this disorder to a mild degree, even when comparing different variants in the same exon of the *LMNA* gene. However, these results should be considered with caution, given the small sample size related to this ultra-rare disease, which is why further studies with a larger number of patients should be carried out. The exact mechanisms of this gene dysregulation, the underlying cause for its tissue-specific effects and the phenotypic and metabolic differences associated with each specific variant are yet to be elucidated.

## Figures and Tables

**Table 1:** Pathogenic variants detected in exon 8 of the *LMNA* gene in the study population.

Pathogenic Variant	Protein	cDNA	Patients (*n* = 47)
N466	p.(Asn466Asp)	c.1396A>G	15
R482	p.(Arg482Trp)	c.1444C>T	30
	p.(Arg482Gln)	c.1445G>A	2

cDNA: DNA change.

**Table 2:** Demographic data, anthropometric measurements, and body composition determined by dual-energy X-ray absorptiometry (DXA) in FPLD2 patients depending on the variant in exon 8 of the *LMNA* gene.

	Patients with the R482 Variant (*n* = 32)	Patients with the N466 Variant (*n* = 15)	*p* Value
Age (years)	51.8 ± 18.1	44.1 ± 19.9	0.283
Gender (*n* women)	24 (75.0%)	11 (73.3%)	0.585
Phenotype onset (*n*)			0.329
Childhood	1 (3.1%)	2 (13.3%)	
Puberty	18 (56.2%)	7 (46.7%)	
Adolescence	2 (6.2%)	3 (20.0%)	
Adulthood	9 (28.1%)	2 (13.3%)	
Unknown	2 (6.2%)	1 (6.7%)	
Height (cm)	164.2 ± 10.5	158.2 ± 14.7	0.317
Weight (kg)	66.5 ± 14.3	59.0 ± 16.1	0.473
BMI (kg/m^2^)	24.4 ± 3.3	23.1 ± 4.6	0.539
Waist perimeter (cm)	82.9 ± 9.9	79.9 ± 11.60	0.718
Hip perimeter (cm)	90.4 ± 9.6	85.7 ± 11.9	0.496
Triceps skinfold (mm)	6.2 ± 3.9	4.9 ± 1.9	0.318
Biceps skinfold (mm)	5.0 ± 2.7	4.8 ± 1.4	0.528
Subscapular skinfold (mm)	22.6 ± 9.9	21.1 ± 11.2	0.266
Suprailiac skinfold (mm)	8.6 ± 3.6	9.9 ± 4.4	0.566
Thigh skinfold (mm)	4.2 ± 1.8	5.6 ± 2.0	0.002 *
Calf skinfold (mm)	3.6 ± 1.6	4.2 ± 1.9	0.318
Total fat (kg)	15.7 ± 3.0	13.9 ± 5.3	0.427
Upper-limb fat (kg)	1.5 ± 0.4	1.5 ± 0.6	0.928
Lower-limb fat (kg)	3.0 ± 0.6	2.7 ± 0.8	0.487
Trunk fat (kg)	9.1 ± 2.0	8.7 ± 3.9	0.880
Visceral fat mass (kg)	1.0 ± 0.3	1.1 ± 0.7	0.657
Visceral fat volume (cm^3^)	1.1 ± 0.3	1.2 ± 0.7	0.658
Total FFM (kg)	48.3 ± 9.5	43.6 ± 12.6	0.548
Upper-limb FFM (kg)	5.1 ± 1.0	5.2 ± 2.0	0.928
Lower-limb FFM (kg)	14.2 ± 2.2	13.6 ± 4.4	0.928
Trunk FFM (kg)	21.9 ± 2.7	21.6 ± 6.0	0.651
Total BMD (g/cm^2^)	1.1 ± 0.1	1.1 ±0.1	0.445
Upper-limb BMD (g/cm^2^)	0.8 ± 0.2	0.8 ± 0.1	0.181
Lower-limb BMD (g/cm^2^)	1.1 ± 0.1	1.1 ± 0.2	0.836
Trunk BMD (g/cm^2^)	0.9 ± 0.1	0.9 ± 0.1	0.907
Spine BMD (g/cm^2^)	1.1 ± 0.1	1.1 ± 0.1	0.628
Pelvis BMD (g/cm^2^)	1.0 ± 0.1	1.0 ± 0.1	0.836

Data are mean ± SD or *n* (%) values. BMI: body mass index; FFM: fat-free mass; BMD: bone mineral density. * *p* < 0.05.

**Table 3:** Clinical data of FPLD2 patients depending on the variant in exon 8 of the *LMNA* gene.

	Patients with the R482 Variant (*n* = 32)	Patients with the N466 Variant (*n* = 15)	*p* Value
Hepatomegaly (*n*)	9 (28.1%)	5 (33.3%)	0.319
Acanthosis nigricans (*n*)	14 (43.8%)	5 (33.3%)	0.363
Acrochordons (*n*)	6 (18.8%)	2 (13.3%)	0.497
Phlebomegaly (*n*)	26 (81.2%)	12 (80.0%)	0.604
Muscle hypertrophy (*n*)	22 (68.8%)	11 (73.3%)	0.516
Muscle pain (*n*)	10 (31.2%)	3 (20.0%)	0.332
Polyphagia (*n*)	8 (25.0%)	1 (6.7%)	0.136
Hepatic steatosis (*n*)	12 (37.5%)	4 (26.7%)	0.349
Pancreatitis (*n*)	0 (0.0%)	3 (20.0%)	0.009 *
DM (*n*)	10 (31.2%)	6 (40.0%)	0.393
DM complications			
Retinopathy (*n*)	1 (10.0%)	1 (16.7%)	0.625
Nephropathy (*n*)	1 (10.0%)	2 (33.3%)	0.304
Neuropathy (*n*)	0 (0.0%)	2 (33.3%)	0.125
Hypertension (*n*)	9 (28.1%)	3 (20.0%)	0.600
Ischemic cardiopathy (*n*)	4 (12.5%)	3 (20.0%)	0.394
Left ventricular hypertrophy (*n*)	2 (6.2%)	1 (6.7%)	0.694
Stroke (*n*)	1 (3.1%)	0 (0.0%)	0.681
Sleep apnoea (*n*)	3 (9.4%)	1 (6.7%)	0.619
PCOS ^†^ (*n*)	5 (21.7%)	3 (30.0%)	0.461
Pregnancy loss ^†^ (*n*)	3 (13.0%)	0 (0.0%)	0.325
Goitre (*n*)	6 (18.8%)	1 (6.7%)	0.270
Malignancy (*n*)	4 (12.5%)	1 (6.7%)	0.483

Data are *n* (%) values. DM: diabetes mellitus; PCOS: polycystic ovary syndrome. ^†^ Among FPLD2 women. * *p* < 0.05.

**Table 4:** Analytical data of FPLD2 patients depending on the variant in exon 8 of the *LMNA* gene.

	Patients with the R482 Variant (*n* = 32)	Patients with the N466 Variant (*n* = 15)	*p* Value
Fasting glucose (mg/dL)	121.8 ± 51.8	131.4 ± 90.3	0.791
HbA1c ^†^ (%)	7.4 ± 2.0	8.2 ± 2.3	0.573
Insulin (mIU/L)	19.4 ± 15.1	17.5 ± 13.7	0.879
HOMA-IR	4.8 ± 2.1	1.4 ± 0.6	0.190
C-peptide (ng/mL)	2.9 ± 0.8	1.6 ± 0.5	0.143
Total cholesterol (mg/dL)	191.8 ± 29.9	166.2 ± 86.6	0.083
LDL-C (mg/dL)	129.8 ± 30.4	73.9 ± 26.0	0.025 *
HDL-C (mg/dL)	36.5 ± 9.9	32.4 ± 5.6	0.755
Non-HDL-C (mg/dL)	161.5 ± 40.6	147.7 ± 87.1	0.429
Triglycerides (mg/dL)	130.0 (55–505)	177.5 (56–1937)	0.029 *
AST (IU/L)	20.5 ± 14.8	24.7 ± 9.4	0.116
ALT (IU/L)	26.2 ± 22.4	29.4 ± 9.4	0.149
GGT (IU/L)	21.2 ± 13.5	23.5 ± 14.2	0.765
Creatinine (mg/dL)	0.7 (0.4–6.9)	0.6 (0.4–1.2)	0.109
CK (IU/L)	121.9 ± 72.5	96.7 ± 41.1	0.720
Leptin (μg/L)	3.3 ± 2.8	2.5 ± 2.1	0.596

Data are mean ± SD or median (IQR) values. HbA1c: glycated hemoglobin; LDL-C: low-density lipoprotein cholesterol; HDL-C: high-density lipoprotein cholesterol; AST: aspartate aminotransferase; ALT: alanine aminotransferase; GGT: γ-glutamyl transpeptidase; CK: creatinine kinase. ^†^ Among patients with diabetes. * *p* < 0.05.

## Data Availability

The data presented in the study are available on request from the corresponding author. These data are not publicly available due to privacy concerns relating to personal clinical and genetic information.
